# Vibrissa growth rate in California sea lions based on environmental and isotopic oscillations

**DOI:** 10.1371/journal.pone.0204641

**Published:** 2018-10-10

**Authors:** Martha P. Rosas-Hernández, Claudia J. Hernández-Camacho, Eduardo González-Rodríguez, David Aurioles-Gamboa

**Affiliations:** 1 Laboratorio de Ecología de Pinnípedos "Burney J. Le Boeuf", Centro Interdisciplinario de Ciencias Marinas, Instituto Politécnico Nacional. La Paz, B.C.S., México; 2 Centro de Investigación Científica y de Educación Superior de Ensenada, Unidad La Paz. La Paz, B.C.S., México; University of California Santa Cruz, UNITED STATES

## Abstract

Pinniped vibrissae provide information on changes in diet at seasonal and annual scales; however, species-specific growth patterns must first be determined in order to interpret these data. In this study, a simple linear model was used to estimate the growth rate of vibrissae from adult female California sea lions (*Zalophus californianus*) from San Esteban Island in the Gulf of California, Mexico. The δ^15^N and δ^13^C values do not display a marked oscillatory pattern that would permit direct determination of the time period contained in each vibrissa; thus, time (age) was calculated in two ways: 1) based on the correlation between the observed number of peaks (Fourier series) in the δ^15^N profile and the length of each vibrissa, and 2) through direct comparison with the observed number of peaks in the δ^15^N profile. Cross-correlation confirmed that the two peaks in the δ^15^N profile reflected the two peaks in the chlorophyll-a concentration recorded annually around the island. The mean growth rate obtained from the correlation was 0.08 ± 0.01 mm d^-1^, while that calculated based on the observed number of peaks was 0.10 ± 0.05 mm d^-1^. Both are consistent with the rates reported for adult females of other otariid species (0.07 to 0.11 mm d^-1^). Vibrissa growth rates vary by individual, age, sex, and species; moreover, small differences in the growth rate can result in significant differences over the time periods represented by the isotopic signal. Thus, it is important to assess this parameter on a species-by-species basis.

## Introduction

Many recent studies have employed stable isotope analysis in order to assess the diet, nutritional status, and feeding ecology of both wild and captive animals [1]. Nitrogen (^15^N/^14^N or δ^15^N) and carbon (^13^C/^12^C or δ^13^C) isotope ratios allow us to infer an organism’s trophic position and potential feeding areas, respectively. This is possible because the tissues of predators are enriched in ^15^N and ^13^C relative to the prey they consume [2–4].

This enrichment allows us to obtain trophic information that can be interpreted at varying temporal scales based on the distinct metabolic or renewal rates of different tissues [5]. For example, the stable isotope ratios in liver represent the diet assimilated during the previous weeks; in contrast, the stable isotope ratios in muscle provide information regarding the diet one to two months prior [6, 7].

The renewal time of a given tissue is determined by its structural organization, cellular composition, and biochemistry [8]. Protein and other tissue components are in a state of dynamic equilibrium involving the continuous degradation of old components and the synthesis of new ones [9–11]. Liver, pancreas, and adipose tissues experience more rapid renewal than connective tissues and bone [6, 7, 12]. However, the renewal rate of metabolically active tissue is difficult to assess as not all of the tissue in a given structure is replaced at once [13]. In contrast, keratin- (e.g., vibrissae, nails, talons, fur) or dentin-based (e.g., tooth) tissues do not undergo any replacement; thus, they constantly grow in length or accumulate new layers, respectively, and therefore are considered accretionary tissues [14, 15].

Accretionary or metabolically inert tissues retain information from the moment they develop, permitting us to track seasonal and interannual changes in feeding [15–17], evaluate the degree of specialization in individual diets, and examine habitat use in marine mammals [18–20]. Depending on its total length and specific growth rate, a single vibrissa can integrate diet information from one or more years [14, 18, 21]. Thus, this tissue is particularly suitable for studies on seasonal variation in feeding.

Vibrissa growth patterns vary for different pinniped species and families. In phocids, vibrissa growth is non-linear (von Bertalanffy model) with the growth coefficient varying from 0.13 to 0.36 d^-1^ in adults [3, 22, 23]. In contrast, for otariids like the California sea lion (*Zalophus californianus;* CSL), vibrissa growth is linear with growth rates ranging from 0.02 to 0.16 mm d^-1^ in adults [23–26]. This considerable interspecific variation in vibrissa growth rates highlights the importance of assessing this parameter on a species-by-species basis. Previous studies have assessed the growth rate using a variety of estimation techniques. In wild pinnipeds, the growth rate has been estimated using ^15^N-enriched glycine as a vibrissa marker to determine the time between successive peaks. The number of oscillations in the isotope profile of a given vibrissa also have been used as an indicator of time (i.e., a certain number of oscillations corresponds to one year). Meanwhile, in captive pinnipeds, the growth rate has been calculated using photogrammetry and direct measurement of vibrissa length [21, 23, 25]. However, animals in captivity are under controlled feeding conditions; thus, their vibrissa growth may differ from that of their counterparts in the wild that are subject to seasonal variation in prey abundance and availability. Photogrammetry may underestimate the growth rate as three-dimensional curved structures become distorted when flattened into two-dimensional photographs, both for wild and captive animals [27]. Moreover, the total vibrissa length can be underestimated due to abrasion from contact with the substrate as this factor is not typically taken into account when measuring vibrissae for photogrammetric analysis [23].

In the present study, we estimated the vibrissa growth rate in wild adult female CSLs in the Gulf of California, Mexico, using the number of oscillations in the δ^15^N profile of a given vibrissa as an indicator of time (a certain number of oscillations corresponds to one year) (S1A Fig). Analysis of the periodicity of the ^15^N isotope profile has been used to examine vibrissa growth in several different species [15, 19, 21, 25, 26]. The oscillations in the values of δ^13^C and δ^15^N reported for these species are primarily influenced by changes in feeding habitats and diet composition, respectively [19, 25]. The specific δ^13^C and δ^15^N values characteristic of the base of the trophic chain in each ecosystem exploited by a given predator allow us to infer potential feeding areas and the trophic position a predator occupies in the trophic chain [28, 29].

In the vibrissae of most pinnipeds, a relatively regular oscillatory pattern has been reported wherein each oscillation represents one year of trophic information [19, 21, 25] (S1B Fig). However, in the present study, it was not possible to measure the vibrissa growth rate by simply using the number of oscillations in the δ^15^N profile as the δ^15^N values of the vibrissae of CSLs from San Esteban Island (SEI) in the central Gulf of California do not display a regular oscillatory pattern, precluding direct determination of the time period contained in each vibrissa (S1A Fig). We believe this is because the area surrounding San Esteban Island (SEI) is very dynamic. Currents and notable changes in the tide level lead to increased CO_2_ concentrations and an influx of nutrients abundant in NO^3-^ that continuously affect the δ^15^N values of phytoplankton [30–32].

To remedy this, we considered the association between the variation in the chlorophyll-a concentration (chlo-a; with two peaks of maximum productivity each year in the study area) and the changes detected in the δ^15^N using the Fourier series to assign time periods to the δ^15^N values and estimate the growth rate based on the linear model [15, 21, 33, 34]. There are two seasonal upwellings (one during winter and the other in the summer) that contribute a considerable amount of NO^3-^ to the system and are reflected in two peaks in the δ^15^N that the Fourier analysis detects as significant changes in the isotope pattern.

It is assumed that female CSLs at this colony do not migrate or move between colonies and therefore changes in isotope values reflect the isotopic composition of the local environment; this assumption is based on the high degree of site fidelity and philopatry exhibited by this species as well as genetic studies conducted at different colonies in the Gulf of California. One study conducted with hot-branded female CSLs from the Los Islotes colony in the southern Gulf of California showed that these animals give birth and nurse their pups at this colony throughout their lives. Moreover, those adult females have never been sighted at other colonies in the Gulf of California during population counts conducted over 25 years [35]. In addition, DNA analysis has shown that CSLs from the Gulf of California are genetically isolated and divided into at least three subpopulations (north, central, and south) [36–38]. The colonies within each subpopulation share biological and ecological characteristics (e.g., trace metal concentration, diet diversity) [38], and each subpopulation is found within one of the four oceanographic zones described by Lluch-Cota [39]. Genetic differentiation and population isolation are the result of the high degree of philopatry and constraints on female dispersal due to the need to return to their breeding colony to nurse their offspring [38]. This has led to the establishment of specific foraging areas over time [38].

## Materials and methods

### Vibrissa sampling and stable isotope analysis

All applicable institutional and national guidelines for the care and use of animals were followed. Animal handling, anesthesia administration, and biological sampling (vibrissa extraction) were approved by the Institutional Animal Care and Use Committee at the Instituto Politécnico Nacional (IPN; National Polytechnic Institute) and by the Secretaría de Medio Ambiente y Recursos Naturales (SEMARNAT; Secretariat of Environment and Natural Resources) under the following research permit: SGPA/DGVS/02012/11. Dr. Carlos R. Sánchez Domínguez (Professional License 2484182) of the Chicago Zoological Society and Brookfield Zoo was responsible for overseeing the administration of anesthesia.

In June 2011, hoop nets were used to capture 11 adult female CSLs on SEI, home to the largest CSL breeding colony in the Gulf of California, Mexico. A mask was used to chemically sedate the animals by administering 5% isoflurane; once the sedative took effect, sedation was maintained with 3% isoflurane. Tweezers were used to extract from the root the longest vibrissa of each female CSL. Care was taken to ensure that all animals were fully recovered from the anesthesia before releasing them on the beach. Immobilization time (total anesthesia time) ranged from 12 to 48 min. The vibrissae were placed in paper envelopes with labels identifying the sample ID and collection date. Samples were transported to the Laboratorio de Ecología de Pinnípedos "Burney J. Le Boeuf" ("Burney J. Le Boeuf" Pinniped Ecology Laboratory) at the Centro Interdisciplinario de Ciencias Marinas (CICIMAR; Interdisciplinary Center for Marine Sciences) in La Paz, Baja California Sur, Mexico.

Once at the laboratory, the total length of each vibrissa was recorded and the cuticle surrounding the root was removed by washing with phosphate-free soap and distilled water to eliminate impurities and lipids. Next, five segments weighing ~1.0 ± 0.2 mg each were cut using a nail clipper; the remaining length of each vibrissa was measured and then this procedure was repeated until the entire vibrissa had been cut into segments in order to later be able to locate each segment along the length of the corresponding vibrissa. Segments weighing ~1.0 ± 0.2 mg were of varying length as this structure is thinner at the tip than at the root. Thus, only the segments closest to the root (with the most recent information) were used as they were of the same length (0.95 ± 0.09 mm); these segments together represent approximately 46 ± 0.07% of the total length of each vibrissa.

Each segment was then placed in a 0.2 ml vial to which a drop of a mixture of chloroform: methanol in a ratio of 2:1 was added to completely remove any residual lipids. Once the solvent had evaporated, the segments were placed in tin capsules for stable isotope analysis. The C and N isotope ratios were determined using the mass spectrometer (Costech 4010 Elemental Analyzer coupled via Conflo III to a Thermo Delta Plus XP [Waltham, MA, USA]) in the Stable Isotope Laboratory at the University of Wyoming, Laramie.

Isotope ratios are expressed as delta values (δ), δ^15^N or δ^13^C = 1000 [(R_sam_/R_std_) - 1], where R_sam_ and R_std_ are the ^15^N/^14^N or ^13^C/^12^C ratios of the sample and the standard, respectively. The units are expressed as parts per thousand (‰). The mass spectrometer had an accuracy of 0.2 ‰ for both δ^13^C and δ^15^N based on well-characterized reference materials from liver (DS_C_ = 0.1, DS_N_ < 0.1), peptone (DS_C_ = 0.4, DS_N_ < 0.1), acetanilide (DS_C_ = 0.1, DS_N_ < 0.1), and alfalfa (DS_C_ = 0.5, DS_N_ < 0.4).

### Harmonic analysis of the δ^15^N isotope profile

Environmental variations (satellite chlo-a) around SEI were used to identify a single year of isotopic information. To obtain these data, satellite images composed of eight days of chlo-a readings taken in 2006–2011 by the MODIS-AQUA sensor level 3 at 4 km^2^ (oceancolor.gsfc.nasa.gov) were used to construct the chlo-a time series and subsequently obtain the periodicity. For each year, a total of 46 weekly mean chlo-a values were obtained; in all, 276 weekly mean values were obtained for the entire study period. To determine the number of oscillation in one year, the time series for both the chlo-a and the δ^15^N values of each vibrissa were evaluated by means of the Fourier harmonic components analysis [40] using the “Periods” technique developed for Matlab. “Periods” is useful for detecting periodicity in time series as the technique is based on determining which components are statistically significant in a time series. Each harmonic (period) obtained is composed of its frequency, phase shift, and amplitude; thus, the sum of all harmonics detected in the analysis provides a model for simulating the original series. The resulting periods are ordered in terms of statistical significance and can be interpreted through association with known environmental events (chlo-a oscillations in this case) [40].

The harmonic adjustment to the δ^15^N profile was applied using the cyclical descent method. The first segment of each vibrissa (i.e., the root) was not included in this analysis because when tested, the δ^15^N value of the initial segment was greater than that of the rest of the vibrissa segments from five different individuals; this notable increase in nitrogen could be erroneously identified as a peak, thus biasing our results.

**Fig 1:**
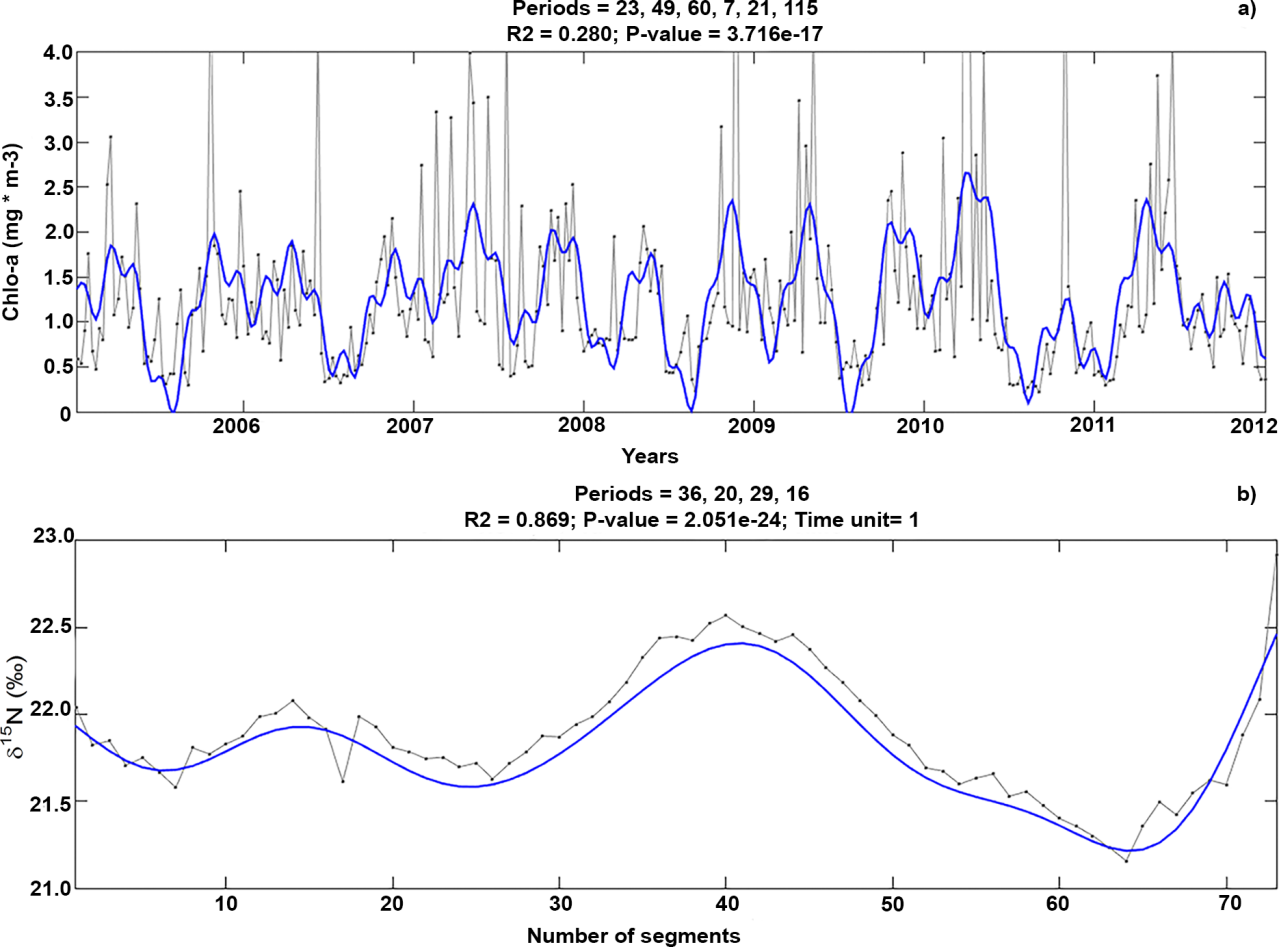
Time series for the chlorophyll-a concentration (chlo-a) over seven years (2005–2012) and for the δ^15^N from one vibrissa. a) Periods detected (23, 49, 60, 7, 21, and 115) in the time series for the chlo-a around San Esteban Island. For the chlo-a, the two main harmonics were located temporally at 23 and 49 weeks; b) Peaks detected (36, 20, 29, and 16) in the time series for δ^15^N in the longest vibrissa in our sample. The resulting number of significant periods in the δ^15^N of each vibrissa is associated with six months of growth.

The two main harmonics in chlo-a were located temporally at 23 and 49 weeks (Fig 1A) and are associated with winter and spring seasonality (in turn associated with upwelling events in the area) and an annual cycle. The most important signal occurred every six months. Based on previous research suggesting that the number of oscillations in the vibrissa δ^15^N profile is associated with the number of annual chlo-a oscillations [15, 19, 21, 25], we hypothesized that the two seasonal peaks in chlo-a would be reflected in two peaks in δ^15^N each year along each vibrissa. Therefore, the resulting number of significant signals (periods in this case) in the δ^15^N of each vibrissa is associated with six months of growth (Fig 1B).

**Fig 2:**
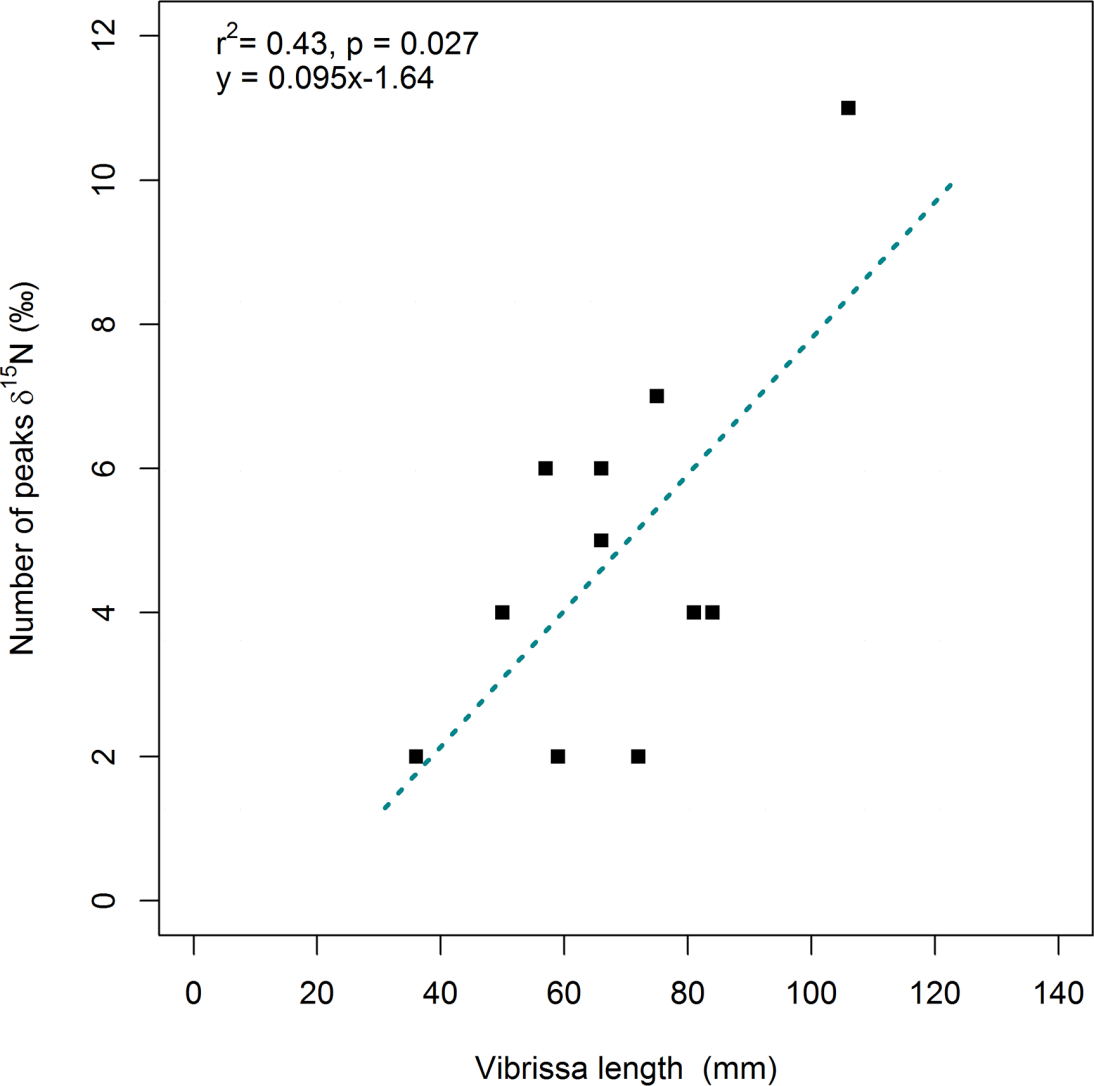
Relationship between length and number of δ^15^N peaks in the vibrissae of adult female California sea lions (*Zalophus californianus*).

A linear regression analysis was performed between vibrissa length (L_t_) and the number of δ^15^N peaks (r^2^ = 0.4, p = 0.027) (Fig 2) to confirm that the number of peaks increased with vibrissa length. There were two δ^15^N peaks each year; thus, it was possible to estimate the age of a given vibrissa based on the expected number of peaks (Table 1). The age of each vibrissa was also estimated using the observed number of δ^15^N peaks; this particular method of estimating age also permitted us to take into account interindividual variability in growth rates.

**Table 1:** Expected (E) and observed (O) number of δ^15^N peaks and time represented by the isotope profile of each vibrissa (ID) collected from adult female California sea lions.

ID	Total Length (mm)	Length Analyzed (mm)	Segments (n)	δ^15^N peaks		Time (days)		Growth Rate (mm d^-1^)	
				O	E	O	E	O	E
1	118	36	35	2	1.8	365	322	0.10	0.11
2	119	75	72	7	5.4	1278	994	0.06	0.08
3	127	57	65	6	3.7	1095	684	0.05	0.08
4	132	50	45	4	3.1	730	563	0.07	0.09
5	152	59	55	2	3.9	365	718	0.16	0.08
6	154	66	60	6	4.6	1095	839	0.06	0.08
7	154	72	71	2	5.2	365	943	0.20	0.08
8	161	66	55	5	4.6	913	839	0.07	0.08
9	165	81	73	4	6.0	730	1098	0.11	0.07
10	175	84	72	4	6.3	730	1149	0.12	0.07
11	199	106	110	11	8.4	2008	1529	0.05	0.07
Mean	150.5 ± 25	68.4 ± 18.7	64.8 ± 19.4	4.8 ± 2.7	4.8± 1.8	879±488	880±322	0.10 ± 0.05	0.08 ± 0.01

### *Zalophus californianus* vibrissa growth rate

We estimated the vibrissa growth rate (*m*) using the linear model where the relationship between length and time is given by:

$$L_{t}=m_{t}t$$ (1)

Where *L*_*t*_ is the length at time *t*, *t* is the age of the vibrissa (based on the expected and the observed number of δ^15^N peaks) (Fig 2), and *m* is the growth rate (unknown). To obtain *m*, we solved the equation as follows:

$$m=L_{t}t^{-1}$$ (2)

The observed growth rate is the growth rate obtained using the observed number of peaks, and the expected growth rate is that based on the expected number of peaks.

### Relationship between environmental parameters and vibrissa isotope profiles

The corresponding chlo-a values around SEI on the dates assigned to each vibrissa segment were then obtained. Based on the time series for both the N stable isotopes and the chlo-a, the mismatch between the environmental variable and the isotopic signal was computed using cross-correlation. Cross-correlation is a useful method for calculating the degree and strength of the association between two time series, as well as the direction of that association [41].

## Results

### *Zalophus californianus* vibrissa growth rate

The total length of each vibrissa analyzed ranged from 36 to 106 mm; depending on total vibrissa length, 35 to 110 segments were analyzed per vibrissa. A total of 713 segments measuring approximately 0.95 ± 0.09 mm each were analyzed. The mean expected growth rate was 0.08 ± 0.01 mm d^-1^, while the mean observed growth rate was 0.1 ± 0.05 mm d^-1^. The interindividual variation was lower for the expected growth rates, which ranged from 0.07 to 0.11 mm d^-1^. Thus, vibrissa age varied from 0.9 to 4.2 years based on the expected number of δ^15^N peaks, and from 1 to 5.5 years based on the observed number of δ^15^N peaks (Table 1). Only seven vibrissae displayed the number of peaks expected based on their length; the remaining four vibrissae had fewer peaks than expected (Fig 2).

### Relationship between environmental parameters and vibrissa isotope profiles

**Fig 3:**
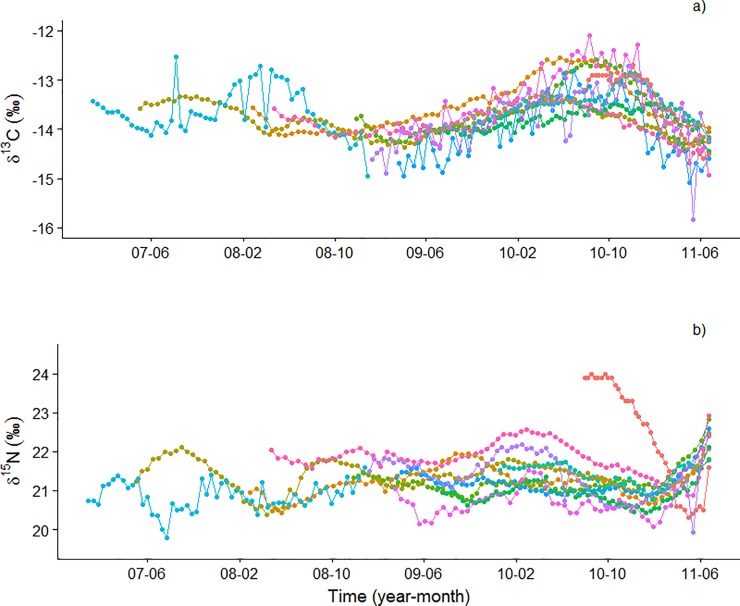
δ^13^C and δ^15^N isotope profiles over time in vibrissae from adult female California sea lions from San Esteban Island, Gulf of California.

The mean values of δ^13^C and δ^15^N obtained from the vibrissae were -13.7 ± 0.4 ‰ and 21.2 ± 0.5 ‰, respectively (S1 Table). No defined oscillatory pattern was observed in either the C or the N isotope profiles (Fig 3). However, “Periods” detected two peaks or changes in the δ^15^N profiles and we associated them with seasonal changes in the chlo-a in the area around SEI; thus, two peaks correspond to a year of isotopic information (Table 1). For example, for Vibrissa 6 (66 mm), 4.6 peaks were expected in its N isotope profile, corresponding to an approximate age of 2.3 years (Table 1); while, six peaks were actually identified in its N isotope profile, corresponding to an approximate age of 3.0 years (Table 1).

### Correlation between the chlo-a and the vibrissae isotopic signal

The results of the cross-correlations (mismatches) were expressed in terms of days in order to visualize the response of the vibrissa isotopic signal to variation in the chlo-a. Based on the linear growth model, an optimal mismatch in the dates represented by the vibrissa segments examined was identified in six vibrissae when the expected growth rate was used and in five vibrissae when the observed growth rate was used. The mismatch between the chlo-a and the δ^15^N was 36 ± 20 days (1.2 months) and 27 ± 17 days (0.9 months), respectively.

## Discussion

### *Zalophus californianus* vibrissa growth rate

Using vibrissae to examine changes in diet requires knowledge of growth patterns [21]. The segments the tissue is divided into must be translated into increments of time in order to associate isotope values with specific time periods [15]. Thus, the designation of an appropriate unit of time to a vibrissa segment is very important as it is the basis for different biological and/or ecological interpretations.

Phocid vibrissa growth differs from that of otariids as their molting and retention periods are distinct. For phocids, like the harbor seal (*Phoca vitulina*) and the leopard seal (*Hydrurga leptonyx*), vibrissa growth is nonlinear [15, 21, 33]; in contrast, for otariids, like the Antarctic fur seal (*Arctocephalus gazella*), the Steller sea lion (*Eumetopias jubatus*), and the CLS (*Z*. *californianus*) in captivity, vibrissa growth is linear [19, 21, 23, 42]. Growth rates may not be linear throughout the entire life cycle as otariid vibrissae grow faster during the juvenile phase; however, once an otariid reaches adulthood, vibrissa growth becomes linear [33]. Thus, it is recommend that a linear model be used for the part of each vibrissa corresponding to the adult phase, as was the case in the present study.

Our estimates of the expected and observed vibrissa growth rates in wild adult female CSLs were 0.08 ± 0.01 mm d^-1^ and 0.10 ± 0.05 mm d^-1^, respectively; both fall within the range reported for other female otariids (0.07 to 0.11 mm d^-1^) even though only 46 ± 0.07% of the total vibrissa length (35 to 110 segments per vibrissa) was used in our analysis [19, 25, 26, 42]. Other estimates based on CSLs in captivity include 0.07 ± 0.04 mm d^-1^ for vibrissae > 100 mm and 0.02 ± 0.03 mm d^-1^ for vibrissae < 100 mm [23]. Our estimated vibrissa growth rates for wild adult female CSLs (118–199 mm total length) (Table 1) were slightly higher and, in the case of the expected growth rates, less variable than that for vibrissa > 100 mm from captive CSLs. The difference in the mean growth rate of vibrissae > 100 mm and our expected estimate (0.08 ± 0.01 mm d^-1^) for vibrissae of similar length was only 0.01 mm d^-1^, which was equal to the interindividual variation estimated in our growth rates. Meanwhile, the difference relative to the observed estimate (0.10 ± 0.05 mm d^-1^) was 0.03 mm d^-1^.

When calculating the time required for a vibrissa to reach a certain length, these differences are more evident particularly when we use the observed growth rate. For example, a 30 mm segment corresponds to 429 days (SD 273–1,000 days) using a growth rate of 0.07 ± 0.04 mm d^-1^, 375 days (SD 333–429 days) using a growth rate of 0.08 ± 0.01 mm d^-1^, and 300 days (SD 200–600) using a growth rate of 0.10 ± 0.05 mm d^-1^, a difference of approximately two to four months. This demonstrates how an apparently small difference in the growth rate for individuals of the same species can lead to a significant difference in the time periods represented by the isotopic signal. It is possible that the differences in the vibrissa growth rates for CSLs in captivity *vs*. their counterparts in the wild are due to the fact that the estimate for captive CSLs was based on both males and females (four females, one male), while our estimate for CSLs in the wild was based only on females. The growth rates for CSLs in captivity were reported along with the total length of the vibrissa but not the sex of the individual [23]. This difference may also be because the method used to estimate the growth rate was not the same (photogrammetry for captive CSLs *vs*. the number of oscillations in the δ^15^N profile for wild CSLs). Moreover, the study on captive CSLs calculated a mean growth rate based on vibrissae from only five individuals, whereas the mean growth rates estimated in this study were based on vibrissae from 11 individuals (one growth rate per individual).

Even within a single species, growth rates vary by sex and age [21, 25, 42]. For example, in otariids (e.g., Antarctic fur seal, Subantarctic fur seal [*A*. *tropicalis*], CSL, Steller sea lions), the vibrissa growth rate is faster for males (0.14 ± 0.02 mm d^-1^) than females (0.08 ± 0.02 mm d^-1^) [19, 21, 23, 25]. Some researchers have argued that the longer vibrissae on males of the genus *Arctocephalus* constitute a secondary sex characteristic that is determined by their faster vibrissa growth rate [25]. However, the authors do not explain how this characteristic would be favored by sexual selection. In Steller sea lions, vibrissae grow faster in immature and subadult individuals (0.2 to 0.33 mm d^-1^) than in adults (0.14 mm d^-1^) [42].

Both of the approaches used to estimate growth rates (expected and observed) in the present study are valid and neither is better than the other one. However, the observed growth rate reflects individual variability that can be interpreted either as isotopic changes at the base of the trophic chain or as changes in diet composition. The observer error associated with sampling may also affect growth rate estimates, although this error should be minimal considering that: 1) the vibrissae were only sampled from adult females, 2) the vibrissae were collected from individuals in the same colony over a short period of time, and 3) only the longest vibrissa from each individual was analyzed. Other researchers have argued that the interindividual variation in growth may be due to differences in the length of the vibrissae analyzed [21, 25].

### Relationship between environmental parameters and vibrissa isotope profiles

The “Periods” technique is a useful tool for detecting the frequency of variation (peaks) in δ^15^N values where well-defined oscillatory patterns in the vibrissa isotope profile are lacking, as in this case study [19, 25]. Based on our analysis, we inferred changes in the ^15^N isotope profile in order to then compare them with changes over time in the chlo-a in the feeding area.

The variability in the number of peaks identified in the vibrissa may be due to factors that influence δ^15^N values like changes in diet, feeding area, and the isotopic baseline of the trophic chain. However, it is difficult to determine which of these factors contributed to the peaks as they did not display an oscillatory pattern in our vibrissae.

Ecological and physiological processes can affect the isotopic signature in animals. Many marine mammals experience cycles of feeding and energy expenditure that can alter their isotopic signatures and the ecological interpretation of the data [43]. For example, territorial male CSLs fast at the beginning of the reproductive cycle; while fasting, males rely on their energetic reserves the consumption of which can be reflected in an increase in the δ^15^N values in keratinous tissues while the δ^13^C values remain the same [44, 45].

In females, gestation and lactation can cause a decrease in δ^15^N values, while δ^13^C values are unaffected [45]. However, other studies suggest the nutritional and reproductive physiology have no effect on the isotopic signature of the vibrissae from females of different otariid species due to the fact that: 1) the δ^13^C and δ^15^N values for vibrissae from females covary synchronically [25], 2) isotopic cycles also have been reported in vibrissae from males of different otariid species [21, 25], and 3) the isotopic oscillations in vibrissae from females of two sympatric fur seal species (*A*. *gazella* and *A*. *tropicalis*) have the same periodicity even though their lactation periods vary considerably (four and 10 months, respectively) [25].

We interpret the oscillations (peaks) in the isotopic signatures of the vibrissae from the wild adult female CSLs we sampled as reflecting changes in the chlo-a concentration around the colony rather than a shift in feeding area; the isotopic signals of vibrissae from females exhibit low interindividual variability (13.7 ± 0.4 ‰ δ^13^C and 21.2 ± 0.5 δ^15^N) suggesting a certain degree of specialization in feeding areas, as has been reported for CSLs in California [46]. However, four female CSLs presented fewer δ^15^N peaks than expected as a result of the environmental variation of the area, suggesting that their preferred feeding area may change over time. For example, lactating female CSLs on Granito Island (132 km north of SEI) show greater interindividual variability in their isotope values, diving parameters, and feeding areas during the warm season (July-August, less productive) relative to the cold season (February-March, more productive) [47]. During the warm season, resource availability is limited and therefore intraspecific competition increases, forcing female CSLs to move to other areas. Moreover, foraging patterns in lactating female CSLs are different during the breeding season *vs*. during the post-breeding season on San Miguel Island off the coast of California, USA, possibly due to seasonal changes in prey distribution [48].

### Correlation between the chlo-a and the vibrissa isotopic signal

The correlation between the variability of the chlo-a and that of the δ^15^N isotopic signal revealed a 1.2 and 0.9 months mismatch depending on the growth rate used (expected and observed respectively), to assign dates to the vibrissa segments such that the chlo-a was reflected in the CSL vibrissa isotopic signal either 1.2 or 0.9 months later depending on the approach used. This mismatch is due to the fact that as energy flows through different levels of the trophic web [49, 50], it is not immediately reflected in the isotopic signal of a top predator. When there is a change in the phytoplankton δ^15^N as a result of environmental variation in the ecosystem, these changes are only reflected in the zooplankton (next trophic level) after a certain amount of time; they are later reflected in fish after yet another period of time, and so on until reaching the top trophic levels [51, 52]. The growth rate for the phytoplankton population is approximately 1.2 d^−1^ or more; thus, their stable isotope signal represents the capture of N from sources consumed during the last few days, whereas the growth rate for zooplankton ranges from days to weeks [52].

## Conclusions

For most of the vibrissae from wild adult female CSLs from SEI in the central Gulf of California, Mexico, a peak in the δ^15^N profile represented six months of vibrissa growth. The approach used in this study to estimate the growth rate is recommended for species or populations that lack an annual periodicity in their N isotope values. It is still unclear whether all CSLs throughout the species’ entire distribution also lack an annual periodicity; thus, this approach only applies to the population of CSLs from the central Gulf of California. The growth rates obtained here using the linear model are consistent with those reported for adult females of this and other otariid species. Although the pattern of vibrissa growth is similar for all otariids (linear), we recommend that the age- and sex-specific growth rates be determined for each species and individual whenever possible. Moreover, researchers also should consider whether animals are captive or wild in order to more accurately assign periods to the isotopic information contained in their vibrissae.

## Supporting information

S1 Fig **δ^15^N profiles for (a) the California sea lion and (b) the Antarctic fur seal [taken from Cherel, Kernaléguen (19)].** For the Antarctic fur seal, oscillations are consistent along the length of each vibrissa and each oscillation corresponds to one annual cycle [19]. Meanwhile, for the California sea lion there is no clearly defined oscillatory pattern. a) δ^13^C (filled circles) and δ^15^N (open squares) values along the length of a vibrissa from a female California sea lion in our study. b) δ^13^C (filled circles) and δ^15^N (open diamonds) values along the length of a vibrissa from a male Antarctic fur seal. Dotted lines indicate the isotope estimates for fronts and water masses (APF: Antarctic polar front; STF: subtropical front).
(TIF)

S1 Table δ^15^N values for each vibrissa segment. (XLS)
