# Supplementary figures and images for: Vibrissa growth rate in California sea lions based on environmental and isotopic oscillations
Source: PLoS One. 2018 Oct 10;13(10):e0204641. doi: 10.1371/journal.pone.0204641 (PMC6179384; doi:10.1371/journal.pone.0204641)

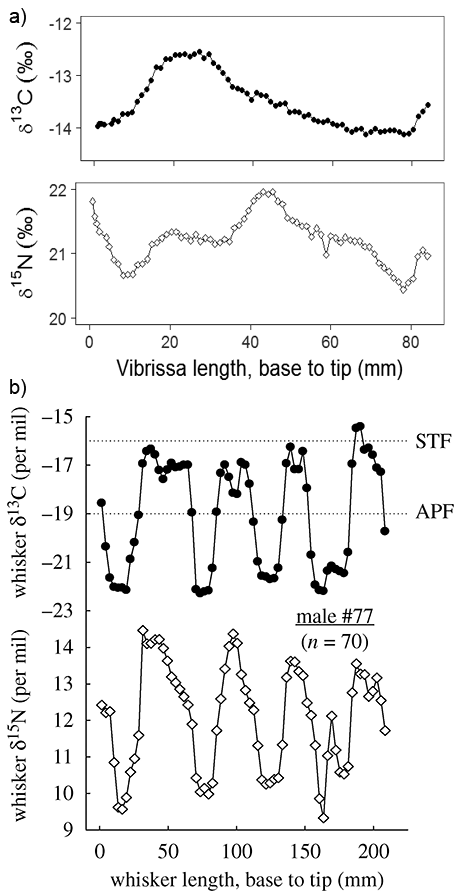

Supplement: S1 Fig — δ15N profiles for (a) the California sea lion and (b) the Antarctic fur seal [taken from Cherel, Kernaléguen (19)]. For the Antarctic fur seal, oscillations are consistent along the length of each vibrissa and each oscillation corresponds to one annual cycle [19]. Meanwhile, for the California sea lion there is no clearly defined oscillatory pattern. a) δ13C (filled circles) and δ15N (open squares) values along the length of a vibrissa from a female California sea lion in our study. b) δ13C (filled circles) and δ15N (open diamonds) values along the length of a vibrissa from a male Antarctic fur seal. Dotted lines indicate the isotope estimates for fronts and water masses (APF: Antarctic polar front; STF: subtropical front). (TIF) [file pone.0204641.s001.tif]
